# A Rare Presentation of Drug-Induced Autoimmune Hepatitis and the Role of Male Enhancement Supplements

**DOI:** 10.7759/cureus.51770

**Published:** 2024-01-06

**Authors:** Juan Enciso, Ruhi Vasavada-Patel, Kyle Lien

**Affiliations:** 1 Internal Medicine, University of South Florida Morsani College of Medicine, Tampa, USA

**Keywords:** over-the-counter supplement, aih, aih -autoimmune hepatitis, type i aih, autoimmune hepatitis, type 1 drug-induced autoimmune hepatitis, diaih, drug-induced autoimmune hepatitis

## Abstract

Autoimmune hepatitis (AIH) is a condition characterized by an autoimmune response resulting in chronic inflammatory liver disease. Its presentation is marked by significant increases in serum immunoglobulins and the production of active autoantibodies that target liver tissue. AIH is often associated with other autoimmune disorders, which can lead to overlapping clinical syndromes. However, alternative theories propose that exposure to specific environmental triggers can initiate this autoimmune cascade. We present the case of a 45-year-old male who sought evaluation for abdominal discomfort and was subsequently diagnosed with drug-induced AIH (DIAIH) following prolonged use of an over-the-counter male-enhancing supplement.

## Introduction

Autoimmune hepatitis (AIH) is an autoimmune inflammatory condition characterized by the production of certain autoantibodies and serum hyperimmunoglobulinemia that specifically targets healthy liver tissue. However, the disease pathogenesis and causation mechanism are still not fully understood. Initially, AIH may present as an acute condition, which can progress into a chronic and slowly evolving inflammatory process, eventually leading to chronic liver disease and cirrhosis. AIH can manifest at any age and within various racial groups, but it is more prevalent among women [1]. Although the pathogenesis of this process remains unclear, current theories suggest the involvement of environmental triggers via a supposition as an autoantigens along with major histocompatibility complex (MHC) activation and T-cell-mediated receptor activation involvement [2]. Additionally, alternative hypotheses propose that the disease's nature may be influenced by specific environmental triggers acting on individuals with a genetic predisposition for autoimmune processes, setting off a cascade of events leading to autoimmune liver injury [3]. These environmental triggers may encompass viral infections and exposure to certain medications, with the most common culprit medications including antibiotics such as minocycline and nitrofurantoin, which are implicated in 90% of cases [4-6].

Differentiating the clinical manifestation of Drug-Induced AIH (DIAIH) from Drug-Induced Liver Injury (DILI) holds crucial importance, mainly due to the histological resemblance of DILI to AIH, leading to the proposition that DIAIH is a variant of DILI [7]. In instances where a comprehensive social review aligns with elevated IgG levels, a diagnosis can be established based on clinical indications without the need for a liver biopsy. However, when the clinical history or illness presentation lacks clarity regarding the underlying cause of liver injury, a liver biopsy becomes essential for further differentiation and accurate diagnosis.

This case report was previously presented as a meeting poster at the 2022 Society of General Internal Medicine (SGIM) Annual Scientific Meeting on April 6, 2022.

## Case presentation

**Figure 1:**
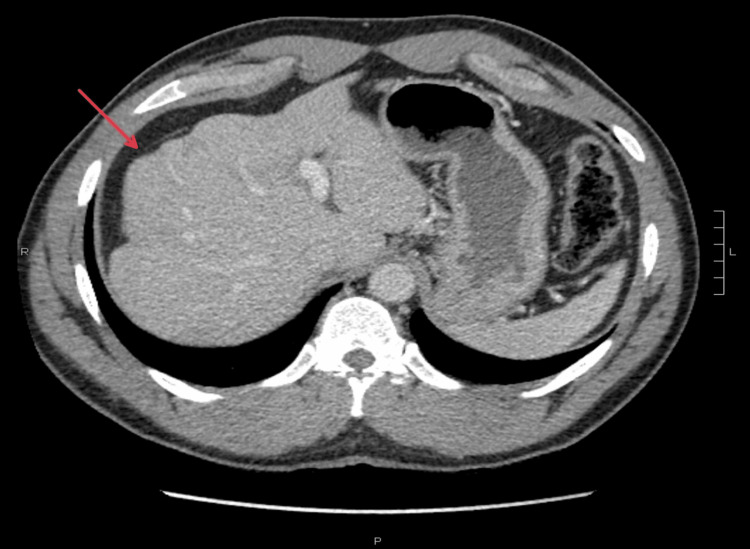
CT of the abdomen showing nodular liver contour with capsular retraction concerning for cirrhosis.

A 45-year-old male with a medical history of hypertension and hyperlipidemia presented with progressive right upper and lower quadrant abdominal discomfort with symptom onset three days prior. The patient reported no active drug use or history of additional over-the-counter (OTC) medications or supplements on assessment. He reported a moderate alcohol history limited to binge drinking episodes of 12-14 beers on the weekends and occasional one beer after work on the weekdays. On admission, lab results were notable for elevated aspartate aminotransferase (AST) of 463, peaking at 1110 (normal value <36 U/L), and alanine aminotransferase (ALT) of 680, peaking at 1771 (normal ALT < 26 U/L), alkaline phosphatase of 119, peaking at 171 (normal level < 147 U/L), and total bilirubin of 1.8 (normal level < 1.3mg/dL). The acute viral hepatitis panel (hepatitis A, B, C, D, and E), cytomegalovirus (CMV), Epstein-Barr virus (EBV), antinuclear antibody (ANA), ceruloplasmin, and serum acetaminophen were negative. Iron studies revealed elevated serum iron: 259 (normal 60-170 mcg/dL), percent iron saturation: 90.9% (normal 20% to 50%), and ferritin: 1652.8 (normal 24-336 mcg/L) with a total iron-binding capacity (TIBC) of 310 (normal 240 to 450 mcg/dL). Preliminary imaging, including CT with IV contrast of the abdomen and pelvis, revealed a nodular liver contour concerning undiagnosed liver cirrhosis (Figure 1). Ultrasonography (US) of the liver with Doppler was negative for hepatic vascular occlusion and bile duct dilation. 

**Figure 2:**
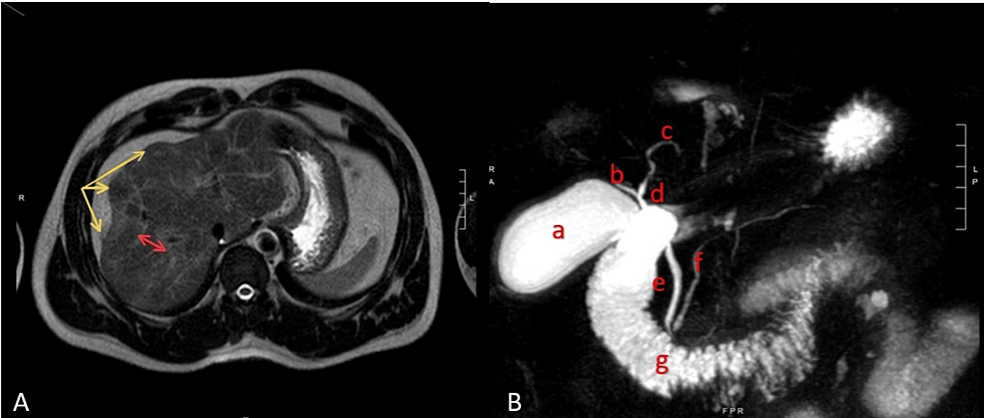
(A) MRI images showing confluent fibrosis in Segment VIII (red arrow) and lobulated liver contour (yellow arrows). (B) MRCP image showing gallbladder (a), right hepatic duct (b), left hepatic duct (c), common hepatic duct (d), common bile duct (e), pancreatic duct (f), and duodenum (g). The cystic duct is obscured by the small intestine. MRCP: Magnetic resonance cholangiopancreatography

Despite an initial absence of significant social and pharmacological indicators, concerns arose regarding potential supplementary factors based on the patient's physique and muscular build, coupled with abnormal preliminary liver function studies. These observations and suspicions prompted an investigation into the possibility of acute liver injury resulting from either drug-induced or autoimmune processes. Further workup, including IgG levels, was reported at 3399 g/L (normal range 6.0 - 16.0 g/L), and an autoimmune workup was positive, including a positive antimitochondrial antibody (AMA) and an anti-smooth muscle antibody (ASMA) level of 63 (normal range - undetectable). Additional imaging, including a magnetic resonance cholangiopancreatography (MRCP), showed lobulated hepatic contour without steatosis, iron deposition, and confluent fibrosis (Figure 2A-2B).

**Figure 3:**
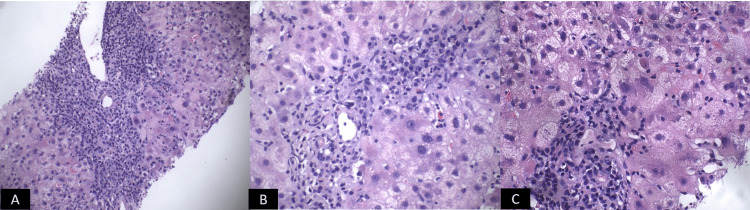
(A-C) Liver biopsy showing lymphocyte-predominant lobular disarray and portal inflammation with lymphoplasmacytic infiltrate and eosinophils. No evidence of PAS-positive/diastase-resistant globules, portal neutrophils, or significant fibrosis, suggesting an autoimmune process. PAS: Periodic-acid Schiff stain

Subsequently, a trans-jugular liver biopsy was remarkable for lymphocyte-predominant lobular disarray and portal inflammation with lymphoplasmacytic infiltrate and eosinophils. There was no evidence of parenchymal or mesenchymal iron deposition, periodic-acid Schiff stain (PAS)-positive/diastase-resistant globules, portal neutrophils, or significant fibrosis, suggesting an autoimmune process (Figure 3A-3B). 

After diagnosis, the patient confided in privacy that he had been taking an over-the-counter 'maximum strength' male enhancement supplement called ExtenZe (Biotab Nutraceuticals, Inc., Monrovia, California, United States) (a herbal nutritional male-enhancer containing dehydroepiandrosterone (DHEA) in addition to a multitude of natural supplements including pregnanolone, ginseng, horny goat weed, etc.) for about one month before admission but increased to maximum dose one week before his presentation. Based on the histopathological findings of lymphoplasmacytic predominance and eosinophilic presence, along with the patient's social history of new OTC supplementation, these findings were suggestive of an autoimmune process due to the increased dosage of the ExtenZe OTC supplement. The patient was promptly started on a high-dose steroid regimen and exhibited notable improvements in liver function test (LFT) results. He was subsequently discharged with a continued steroid taper and a scheduled follow-up with outpatient hepatology.

## Discussion

As stated earlier, epidemiological evaluation for the prevalence of AIH as a female-predominant disorder associated with the conjunction of a wide array of autoimmune confounders and a possible environmental trigger mechanism [1,2]. Its initial presentation can be that of an acute hepatitis process with elevated AST and ALT levels exceeding 10 to 20 times the upper limit of normal with a ratio of alkaline phosphatase presenting at a 1:5 to 1:10 ratio [8]. One key characteristic that helps clue into an autoimmune-mediated process is the presence of elevated gamma globulins, specifically IgG, with normal levels of IgA and IgM [9]. Autoantibodies are also a vital characteristic of this autoimmune process. Different autoantibody subtypes help distinguish between the two possible subtypes of AIH: Type 1 AIH is characterized by the presence of antinuclear antibodies (ANAs), ASMAs, AMAs, anti-soluble liver antigen/liver pancreas antibody-antigen (anti-SLA/LP), anti-single-stranded and anti-double-stranded DNA, and atypical perinuclear anti-neutrophil cytoplasmic antibodies [10,11]. Type 2 AIH is characterized by the presence of antibodies to liver-kidney microsome type 1 (LKM-1) alone or accompanied by anti-liver cytosol antibody-1 (ALC-1) [10,11]. 

In addition to the association between autoantibodies and hyperglobulinemia, histological criteria are essential in diagnosing AIH. The predominant presence of mononuclear cells, primarily lymphocytes with occasional eosinophils, surrounding the portal triad and the biliary tree, as well as the presence of biliary architectural alterations (either destructive or nondestructive), represent some of the non-specific histological features associated with this condition [10-12]. 

When suggestive features of AIH are evident, it's important to note that pathological findings characterized by a predominance of lymphoplasmacytic cells may indicate an autoimmune process with AIH components. At the same time, eosinophilia may suggest the involvement of DILI [5]. When both patterns are observed simultaneously, a diagnosis of DIAIH should be considered. The differentiation between AIH and DILI can be challenging due to the influence of various confounding biological agents that can lead to these clinical outcomes. Notably, oxyphenisatin, nitrofurantoin, minocycline, clometacin, and alpha-methyl dopa have been associated with developing DIAIH [5,6].

Accurate differentiation between DIAIH and AIH or DILI is crucial in shaping appropriate treatment approaches. AIH typically demands lifelong immunosuppressive therapy. DIAIH can often be managed by discontinuing the triggering agent and attaining complete remission with a six-month steroid taper therapy, eliminating the need for lifelong immunosuppressive regimens [13].

## Conclusions

This case underscores a suspected Type 1 DIAIH resulting from OTC male sexual-enhancing supplements. This presentation suggests an underlying metabolic conversion of specific metabolites into neoantigens, triggering an inflammatory response that ultimately leads to self-inflicted damage.

Accurate diagnosis of DIAIH and its distinction from AIH or DILI is crucial for guiding effective treatment strategies. Whereas AIH typically necessitates lifelong immunosuppressive therapy, management of DIAIH involves discontinuation of the causative agent and supportive therapies. Current literature highlights that individuals diagnosed with DIAIH can attain complete remission through a steroid course and cessation of the responsible agents. Early identification of the implicated substance and swift intervention can mitigate the long-term consequences of the disease and its treatment. It is important to recognize novel agents, such as prescribed medications (minocycline, nitrofurantoin, or alpha-methyl dopa), over-the-counter pharmaceuticals (like ExtenZe and other male enhancement supplements), and even natural supplements are implicated in DIAIH. Staying up-to-date on emerging side effect profiles and ensuring timely and informed therapeutic interventions are paramount. Those diagnosed with DIAIH, like this patient, may achieve remission through a six-month steroid taper therapy, thereby obviating the need for lifelong immunosuppressive regimens.
